# Supplementary material for: Protein Analysis of A. halleri and N. caerulescens Hyperaccumulators When Exposed to Nano and Ionic Forms of Cd and Zn
Source: Nanomaterials (Basel). 2022 Nov 28;12(23):4236. doi: 10.3390/nano12234236 (PMC9736429; doi:10.3390/nano12234236)
Supplement: Supplementary file 1 [file nanomaterials-12-04236-s001.zip › supplementary SM1.pdf]

Supplementary file S1

Protein analysis of *A. halleri* and *N. caerulescens* hyperaccumulators when exposed to nano and ionic forms of Cd and Zn.

Valentina Gallo[1]*, Valentina Serianni*[1]*, Davide Imperiale*[1,2]*, Andrea Zappettini*[3]*, Marco Villani*[3]*, Marta Marmiroli* [1*]*, Nelson Marmiroli* [4]

[1] Department of Chemistry, Life Sciences and Environmental Sustainability, University of Parma, 4 Parma, Italy

[2] Experimental Station for the Food Preservation Industry –Research Foundation, Parma, Italy

[3]Institute of Materials for Electronics and Magnetism (IMEM), National Research Council (CNR), 6 Parma, Italy

[4] The Italian National Interuniversity Consortium for Environmental Sciences (CINSA), Parma, Italy

*Correspondence: marta.marmiroli@unipr.it; Tel.: +39 0521 905698

List of Supplementary material:

Figure S1. Experimental design of *A. halleri*

Figure S2. Experimental design of *N. caerulescens*

Table S3. MapMan BIN assignation and description of differentially abundant proteins in *A. halleri*

Table S4. MapMan BIN assignation and description of differentially abundant proteins in *N. caerulescens*

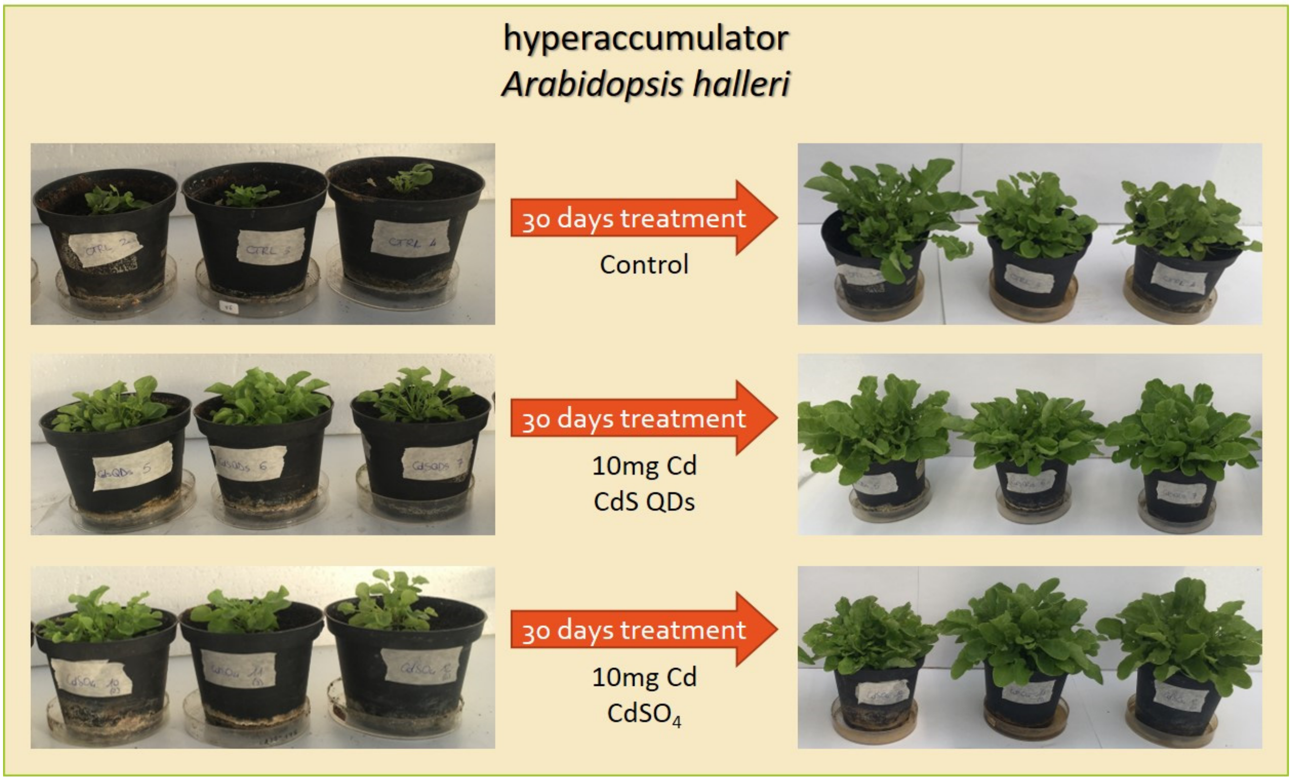

Figure S1. Experimental design. *A. halleri* plants before and after 30 days of treatment with 10 mg of Cd administered in the nano-form of CdS QDs and in the form of $CdSO_4$. Treatment with Cd as CdS QDs (10 mg per pot) had a positive effect on growth of *A. halleri* as compared with the untreated control. Treatment with $CdSO_4$, also had a positive effect.

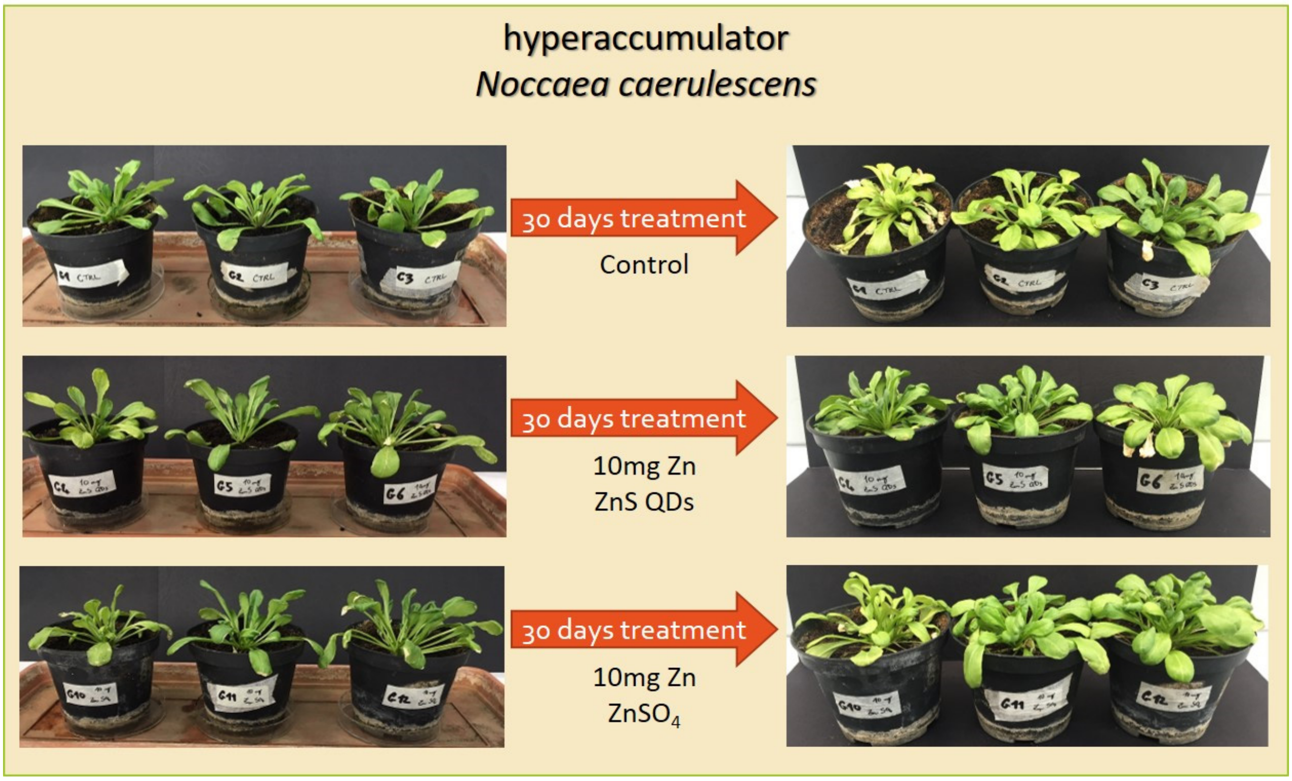

Figure S2. Experimental design. *N. caerulescens* plants before and after 30 days of treatment with 10 mg of Zn administered as ZnS QDs and $ZnSO_4$. *N. caerulescens* treated with either ZnS QDs or $ZnSO_4$ (10 mg per pot) did not show any negative symptoms on the leaf development and color.

Table S3. MapMan BIN assignation and description of differentially abundant proteins in *Arabidopsis halleri*

Protein name	bin code	bin name	description
**14-3-3-like protein B**	16.8.3.1	nucleotide metabolism.synthesis.PRS-PP	
**ABC transporter B family member 28**	29.5.3	transport.ABC transporters and multidrug resistance systems	
**Aldo-keto reductase family 4 member C11**	26.10	minor CHO metabolism.others	Oxidoreductase that may act on a broad range of substrates such as ketosteroids, aldehydes, ketones and sugars.
**ATP synthase delta chain**	1.1.4.7	PS.lightreaction.ATP synthase.delta chain	F_1F_0 ATP synthase produces ATP from ADP in the presence of a proton or sodium gradient. Essential for photosynthesis, probably by facilitating electron transport in both photosystems I and II
**ATP synthase subunit alfa**			Produces ATP from ADP in the presence of a proton gradient across the membrane.
**ATP-dependent Clp protease ATP-binding subunit CLPT1**		protein.targeting.unknown	Accessory protein regulating the assembly of the plastidial Clp protease system
**AT-rich interactive domain-containing protein 5**	20.1.7	RNA.regulation of transcription.AT-rich interaction domain containing transcription factor family	
**BURP domain protein RD22**	11.2.1	stress.abiotic.drought/salt	Acts to suppress chlorophyll degradation under moisture stress.
**Cinnamoyl-CoA reductase 2**	29.5.11.4.2	secondary metabolism.flavonoids.dihydroflavonols.dihydroflavonol 4-reductase	Involved in the formation of phenolic compounds associated with the hypersensitive response. Seems not to be involved in lignin biosynthesis.
**Cysteine proteinase inhibitor 2**	11.9.4.3	protein.degradation.cysteine protease	Specific inhibitor of cysteine proteinases. Probably involved in the regulation of endogenous

			processes and in defense against pests and pathogens
**Cytochrome P450 82G1**	29.5.11.4.3.2	misc.cytochrome P450	Involved in the biosynthesis of homoterpenes, attractants of herbivores parasitoids and predators
**D-2-hydroxyglutarate dehydrogenase**			Catalyzes the oxidation of (R)-2-hydroxyglutarate to 2-oxoglutarate. May be involved in the catabolism of propionyl-CoA derived from beta-oxidation. Involved in degradation of lysine for the supply of carbon and electrons to the ETF/ETFQO complex during dark-induced sugar starvation.
**DEAD-box ATP-dependent RNA helicase 38**	26.9	DNA.synthesis/chromatin structure	ATP-dependent RNA helicase essential for mRNA export from the nucleus. Plays an important role in the positive regulation of CBF/DREB transcription factors.
**Defensin-like protein 249**	30.5	signalling.receptor kinases.S-locus glycoprotein like	
**Defensin-like protein 8**	15.2	stress.biotic.PR-proteins	
**Delta-9 desaturase-like 2 protein**	34.12	lipid metabolism.FA desaturation.desaturase	Involved in lipid metabolism and in polyunsaturated fatty acid biosynthesis.
**DNA repair protein recA homolog 2**	26.9	DNA.synthesis/chromatin structure	Involved in recombination ability and DNA strand transfer activity.
**E3 ubiquitin-protein ligase**	16.2	protein.degradation.ubiquitin.E3.RING	Mediates E2-dependent protein ubiquitination.
**Enoyl-CoA delta isomerase 3**	26.12	lipid metabolism.lipid degradation.beta-oxidation.enoyl CoA hydratase	Able to isomerize both 3-cis and 3-trans double bonds into the 2-trans form in a range of enoyl-CoA species. Essential for the beta oxidation of unsaturated fatty acids.
**F-box protein At3g18320**	29.4	protein.degradation.ubiquitin.E3.SCF. FBOX	

**Glutathione S-transferase U18**	20.1.7	misc.glutathione S transferases	May be involved in the conjugation of reduced glutathione to a wide number of exogenous and endogenous hydrophobic electrophiles and have a detoxification role against certain herbicides.
**Glutathione S-transferase U24**	20.1.7	misc.glutathione S transferases	May be involved in the conjugation of reduced glutathione to a wide number of exogenous and endogenous hydrophobic electrophiles and have a detoxification role against certain herbicides
**GTP-binding protein SAR1A**	1.3.1	signalling.G-proteins	Involved in transport from the endoplasmic reticulum to the Golgi apparatus.
**Heavy metal-associated isoprenylated plant protein 8**	29.4	metal handling.binding, chelation and storage	Heavy-metal-binding protein.
**Metal tolerance protein 1**	27.1	transport.metal	Mediates zinc accumulation in roots and confers resistance to zinc. Involved in sequestration of excess zinc in the cytoplasm into vacuoles to maintain zinc homeostasis. Can also transport cadmium with a low efficiency.
**Nicotinamidase 3**	8.1.7	secondary metabolism.phenylpropanoids	Catalyzes the deamidation of nicotinamide, an early step in the NAD^+ salvage pathway. Prevents the accumulation of intracellular nicotinamide, a known inhibitor of poly(ADP-ribose) polymerases (PARP enzymes).
**Pentatricopeptide repeat-containing protein At1g28690**			
**Pentatricopeptide repeat-containing protein At2g15630**			
**Peroxidase 10**	2.1	misc.peroxidases	Removal of H_2O_2, oxidation of toxic reductants, biosynthesis and degradation of lignin, suberization, auxin catabolism, response to

			environmental stresses such as wounding, pathogen attack and oxidative stress. These functions might be dependent on each isozyme/isoform in each plant tissue.
**Probable protein phosphatase 2C 1**	27.3.6	protein.postranslational modification	May act as negative regulator of GCN5.
**Probable trehalose-phosphate phosphatase 10**			
**Putative defensin-like protein 121**	15.2	stress.biotic.PR-proteins	
**Ras-related protein RABE1d**	26.12	lipid metabolism.lipid degradation.beta-oxidation.enoyl CoA hydratase	Involved in membrane trafficking from the Golgi to the plasma membrane.
**Ribose-phosphate pyrophosphokinase 2**	16.8.3.1	nucleotide metabolism.synthesis.PRS-PP	
**Ribulose bisphosphate carboxylase large chain**	16.4.1	PS.calvin cycle.rubisco large subunit	Involved in the photosynthetic reductive pentose phosphate pathway (Calvin-Benson cycle). Catalyzes the reduction of 1,3-diphosphoglycerate by NADPH
**Serine/threonine phosphatase 2A subunit B' beta isoform**	27.3.6	protein.postranslational modification	The B regulatory subunit may modulate substrate selectivity and catalytic activity, and also may direct the localization of the catalytic enzyme to a particular subcellular compartment. Required for the formation of the PP2A holoenzyme that positively regulates brassinosteroid signaling by dephosphorylating and activating BZR1.
**Sm-like protein LSM1A**	27.1	RNA.processing	Component of the cytoplasmic LSM1-LSM7 complex which is involved in mRNA degradation by promoting decapping and leading to accurate 5'-3' mRNA decay. Required for P-body formation during heat stress

**Succinate dehydrogenase subunit 4**	8.1.7	TCA / organic transformation.TCA.succinate dehydrogenase	Membrane-anchoring subunit of succinate dehydrogenase (SDH).
**Thioredoxin-like 2-1**	21.1	redox.thioredoxin	Thiol-disulfide oxidoreductase that may participate in various redox reactions. Possesses insulin disulfide bonds reducing activity.
**Transcription factor bHLH168**	27.3.6	RNA.regulation of transcription.basic helix-loop-helix family (bHLH)	Plays a role in RNA transcription or processing during stress.
**Transcription factor CPC**	27.3.25	RNA.regulation of transcription.MYB domain transcription factor family	Transcription factor. Determines the fate of epidermal cell differentiation. Represses trichome development by lateral inhibition. Together with GL3 or BHLH2, promotes the formation of hair developing cells (H position) in root epidermis, probably by inhibiting non-hair cell formation. Represses the expression of GL2 and WER in H cells. Positively regulates stomatal formation in the hypocotyl
**Transcription factor KAN4**	27.3.25	RNA.regulation of transcription.MYB domain transcription factor family	Probable transcription factor that regulates carpel integuments formation. Required for the specification of polarity in the ovule inner integument. Modulates the content of flavonols and proanthocyanidin in seeds.
**Tyrosine decarboxylase 1**	16.4.1	secondary metabolism.N misc.alkaloid-like	

Table S4. MapMan BIN assignation and description of differentially abundant proteins in *Noccea caerulescens*

Protein name	bin code	bin name	description
**11-beta-hydroxysteroid dehydrogenase-like 3**	26.22	misc.short chain dehydrogenase/reductase (SDR)	It's an oxidoreductase
**2S seed storage protein 4**	33.1	development.storage proteins	This is a 2S seed storage protein.
**30S ribosomal protein S12, chloroplastic**	29.2.1.2.1.12	protein.synthesis.ribosomal protein.prokaryotic.chloroplast.30S subunit.S12	With S4 and S5 plays an important role in translational accuracy. Located at the interface of the 30S and 50S subunits
**30S ribosomal protein S19, chloroplastic**	29.2.1.2.1	protein.synthesis.ribosomal protein.eukaryotic.40S subunit.S19	Protein S19 forms a complex with S13 that binds strongly to the 16S ribosomal RNA.
**3-ketoacyl-CoA synthase 12**	11.1	lipid metabolism.FA synthesis and FA elongation	Involved in lipid metabolism and fatty acid biosynthesis.
**40S ribosomal protein S3a-1**	29.2.1.2.1.53	protein.synthesis.ribosomal protein.eukaryotic.40S subunit.S3A	
**ACT domain-containing protein ACR7**	13	amino acid metabolism	May bind amino acids.
**Alpha-1,6-mannosyl-glycoprotein 2-beta-N-acetylglucosaminyltransferase**	29.7.8	protein.glycosylation.alpha-1,6-mannosyl-glycoprotein 2-beta-N-acetylglucosaminyltransferase (GnTII)	Catalyzes an essential step in the conversion of oligo-mannose and hybrid to complex N-glycans.

**Arogenate dehydratase/prephenate dehydratase 6, chloroplastic**	13.1.6.3.1	amino acid metabolism.synthesis.aromatic aa.phenylalanine.arogenate dehydratase / prephenate dehydratase	Converts the prephenate produced from the shikimate-chorismate pathway into phenylalanine
**ATP synthase delta chain, chloroplastic**	1.1.4.7	PS.lightreaction.ATP synthase.delta chain	F_1F_0 ATP synthase produces ATP from ADP in the presence of a proton or sodium gradient. Essential for photosynthesis, probably by facilitating electron transport in both photosystems I and II
**ATP-dependent Clp protease proteolytic subunit-related protein 1, chloroplastic**	29.5.5	protein.degradation.serine protease	Required for chloroplast development and differentiation.
**BTB/POZ domain-containing protein At3g50780**	27.3.67	RNA.regulation of transcription.putative transcription regulator	May act as a substrate-specific adapter of an E3 ubiquitin-protein ligase complex (CUL3-RBX1-BTB) which mediates the ubiquitination and subsequent proteasomal degradation of target proteins.
**Calcium-binding protein CML31, probable**	30.3	signanlling. Calcium	Potential calcium sensor.
**Chaperone protein ClpB1**			Molecular chaperone that plays an important role in thermotolerance. Together with HSA32, required for long-term acquired thermotolerance (LAT) in plants and naturally high basal thermotolerance observed in germinating seedlings.
**COP9 signalosome complex subunit 2**	30.11.1	signalling.light.COP9 signalosome	Component of the COP9 signalosome complex (CSN), a complex involved in various cellular and developmental processes such as photomorphogenesis and auxin and jasmonate responses. The CSN complex is an essential

			regulator of the ubiquitin (Ubl) conjugation pathway. It is involved in repression of photomorphogenesis in darkness by regulating the activity of COP1-containing Ubl ligase complexes. The complex is also required for degradation of IAA6 by regulating the activity of the Ubl ligase SCF-TIR complex.
**Cyclin-A3-2**	31.3	cell.cycle	
**Cytochrome b-c1 complex subunit 7-2**	9.5	mitochondrial electron transport / ATP synthesis.cytochrome c reductase	Component of the ubiquinol-cytochrome c oxidoreductase, a multisubunit transmembrane complex that is part of the mitochondrial electron transport chain which drives oxidative phosphorylation. The cytochrome b-c1 complex catalyzes electron transfer from ubiquinol to cytochrome c, linking this redox reaction to translocation of protons across the mitochondrial inner membrane.
**Defensin-like protein 39, putative**	20.1.7.12	stress.biotic.PR-proteins.PR12 (plant defensins)	
**Dihydropyrimidine dehydrogenase (NADP+), chloroplastic**	23.2.1.3	nucleotide metabolism.degradation.pyrimidine.dihydrouracil dehydrogenase	Involved in pyrimidine base degradation. Involved in the recycling of nitrogen from nucleobases to general nitrogen metabolism
**E3 ubiquitin-protein ligase ARI9, probable**	29.5.11.4.2	protein.degradation.ubiquitin.E3.RING	Might act as an E3 ubiquitin-protein ligase, or as part of E3 complex, which accepts ubiquitin from specific E2 ubiquitin-conjugating enzymes and then transfers it to substrates.

**E3 ubiquitin-protein ligase PRT1**	29.5.11.4.3.2	protein.degradation.ubiquitin.E3.SCF.F BOX	E3 ubiquitin-protein ligase that mediates ubiquitination and subsequent proteasomal degradation of target proteins.
**Expansin-A7**	10.7	cell wall.modification	Causes loosening and extension of plant cell walls by disrupting non-covalent bonding between cellulose microfibrils and matrix glucans. No enzymatic activity has been found
**F-box protein At1g61060, putative**	29.5.11.4.3.2	protein.degradation.ubiquitin.E3.SCF.F BOX	
**F-box protein At3g18320**	29.5.11.4.3.2	protein.degradation.ubiquitin.E3.SCF.F BOX	
**F-box/kelch-repeat protein At3g27910, putative**	29.5.11.4.3.2	protein.degradation.ubiquitin.E3.SCF.F BOX	
**FRIGIDA-like protein 3**			
**Heat stress transcription factor B-3**	20.2.	stress.abiotic.heat	Transcriptional regulator that specifically binds DNA sequence 5'-AGAAnnTTCT-3' known as heat shock promoter elements (HSE).
**Jasmonate O-methyltransferase**	17.8.1.1.7	hormone metabolism.salicylic acid.synthesis-degradation.synthesis.methyl-SA methylesterase	Catalyzes the methylation of jasmonate into methyljasmonate, a plant volatile that acts as an important cellular regulator mediating diverse developmental processes and defense responses.
**Late embryogenesis abundant protein 46**	29.4	protein.postranslational modification	Involved dehydration tolerance. Involved in the adaptive response of vascular plants to withstand water deficit. May possess chaperone-like activity under water deficit

**Mitogen-activated protein kinase kinase 7**	30.6	signalling.MAP kinases	May function as a negative regulator of polar auxin transport. Positively regulates plant basal and systemic acquired resistance (SAR).
**NAD(P)H-quinone oxidoreductase subunit K, chloroplastic**	1.3.6	PS.lightreaction.NADH DH	NDH shuttles electrons from NAD(P)H:plastoquinone, via FMN and iron-sulfur (Fe-S) centers, to quinones in the photosynthetic chain and possibly in a chloroplast respiratory chain. Couples the redox reaction to proton translocation, and thus conserves the redox energy in a proton gradient.
**NADH dehydrogenase [ubiquinone] 1 beta subcomplex subunit 7**	23.1.3	nucleotide metabolism.synthesis.PRS-PP	Accessory subunit of the mitochondrial membrane respiratory chain NADH dehydrogenase (Complex I), that is believed not to be involved in catalysis. Complex I functions in the transfer of electrons from NADH to the respiratory chain.
**Nascent polypeptide-associated complex subunit beta**	27.3.67	RNA.regulation of transcription.putative transcription regulator	Part of the nascent polypeptide-associated complex (NAC).
**NDR1/HIN1-like protein 26**	33.2	stress.biotic	Involved in the regulation of sugar, amino acid and some primary metabolite export from companion cells (CCs) to sieve elements (SEs) in phloem. Required for apoplastic phloem sugar loading in source leaves in order to transport it to sink tissues. Required for correct sugar partitioning between source leaves and sink organs.
**Patellin-3**	29.3.4.99	protein.targeting.secretory pathway.unspecified	Carrier protein that may be involved in membrane-trafficking events associated with cell plate formation during cytokinesis. Binds to some

			hydrophobic molecules such as phosphoinositides and promotes their transfer between the different cellular sites.
**Pentatricopeptide repeat-containing protein At2g40720**			
**Pentatricopeptide repeat-containing protein At3g48250**			
**Peroxisomal membrane protein 11-A**	33.1	cell.organization	Involved in peroxisomal proliferation. Promotes peroxisomal duplication, aggregation or elongation without fission
**PHD finger protein ALFIN-LIKE 1**			Histone-binding component, which mark transcription start sites of virtually all active genes
**Phosphatidate cytidylyltransferase 2**	11.3.3	lipid metabolism.phospholipid synthesis.phosphatidate cytidylyltransferase	May be involved in the synthesis of minor phospholipids and in modulation of IP3-mediated signal transduction.
**Probable glutathione S-transferase U19**	26.9	misc.glutathione S transferases	Catalyzes the glutathionylation of 12-oxophytodienoate (OPDA).
**Protein SHI RELATED SEQUENCE 1**			Transcription activator that binds DNA on 5'-ACTCTAC-3' and promotes auxin homeostasis-regulating gene expression (e.g. YUC genes), as well as genes affecting stamen development, cell expansion and timing of flowering. Synergistically with other SHI-related proteins, regulates gynoecium, stamen and leaf development in a dose-dependent manner, controlling apical-basal patterning. Promotes style and stigma formation, and influences vascular development during gynoecium development. May also have a role in

			the formation and/or maintenance of the shoot apical meristem (SAM).
**Protein TIFY 5B**			Repressor of jasmonate responses
**Protein trichome birefringence-like 6**			May act as a bridging protein that binds pectin and other cell wall polysaccharides. Probably involved in maintaining esterification of pectins
**Ras-related protein RABA1c**	30.5	signalling.G-proteins	Intracellular vesicle trafficking and protein transport.
**Ribulose bisphosphate carboxylase large chain**	1.3.1	PS.calvin cycle.rubisco large subunit	Involved in the photosynthetic reductive pentose phosphate pathway (Calvin-Benson cycle). Catalyzes the reduction of 1,3-diphosphoglycerate by NADPH
**Serine carboxypeptidase-like 52, putative**	29.5.5	protein.degradation.serine protease	
**Thioredoxin F1, chloroplastic**	21.1	redox.thioredoxin	Oxidoreductase involved in the redox regulation of enzymes of both reductive pentose phosphate pathway and oxidative pentose phosphate pathway. Under light or reducing conditions, activates in chloroplast the glyceraldehyde-3-phosphate dehydrogenase, the phosphoribulokinase and the fructose-1,6-bisphosphate phosphatase, and inhibits the glucose-6-phosphate dehydrogenase
**Transcription factor bHLH92**	27.3.6	RNA.regulation of transcription.basic helix-loop-helix family (bHLH)	

**Transcription factor IND**	27.4	RNA.RNA binding	Transcription regulator required for seed dispersal. Involved in the differentiation of all three cell types required for fruit dehiscence. Works with ALC, SHP, and FUL to allow differentiation of the lignified valve layer, the spring-loaded mechanism of fruit that promotes opening.
**Transcription factor SRM1**			Transcription activator that coordinates abscisic acid (ABA) biosynthesis and signaling-related genes via binding to the specific promoter motif. Represses ABA-mediated salt stress tolerance. Regulates leaf shape and promotes vegetative growth.
**Transcription initiation factor TFIID subunit 15**	30.5	signalling.G-proteins	TAFs are components of the transcription factor IID (TFIID) complex that is essential for mediating regulation of RNA polymerase transcription.
**tRNA-splicing endonuclease subunit Sen2-1**	27.1.1	RNA.processing.splicing	Constitutes one of the two catalytic subunit of the tRNA-splicing endonuclease complex, a complex responsible for identification and cleavage of the splice sites in pre-tRNA.
**Ubiquinone biosynthesis protein COQ4 homolog, mitochondrial**	9.99	mitochondrial electron transport / ATP synthesis.unspecified	Component of the coenzyme Q biosynthetic pathway. May play a role in organizing a multi-subunit COQ enzyme complex required for coenzyme Q biosynthesis.
**Ubiquitin domain-containing protein DSK2a**	29.5.11.1	protein.degradation.ubiquitin.ubiquiti n	Binds and presumably selects ubiquitin-conjugates for destruction. Acts as a ubiquitin receptor that associates with the 26S proteasomal docking subunit RPN10 for the indirect recognition of ubiquitinated substrates of ubiquitin/26S proteasome-mediated proteolysis (UPP).

**Uncharacterized mitochondrial protein AtMg00280**	1.3.1	PS.calvin cycle.rubisco large subunit	Seems to correspond to a fragment of a former RuBisCO gene.
**Vacuolar protein sorting-associated protein 52**	31.4	cell.vesicle transport	Acts as component of the GARP complex that is involved in retrograde transport from early and late endosomes to the trans-Golgi network (TGN). The GARP complex facilitates tethering as well as SNARE complex assembly at the Golgi.<br>Required for pollen tube elongation and other polar growth
**Vascular-related unknown protein 3**			Involved in the regulation of plant growth.
**Vesicle transport v-SNARE 12**	31.4	cell.vesicle transport	Together with either SYP41 or SYP61, required for membrane fusion; the fusion of phospholipid vesicles containing SYP41 or SYP61 and VTI12 is triggered by YKT61 and YKT62.<br>Functions as a v-SNARE responsible for the docking or fusion of transport vesicles within the trans-Golgi network (TGN) and mediates liposome fusion. Necessary to deliver proteins to the protein storage vacuole (PSV).<br>May be also involved in retrograde traffic to the cis-Golgi
**YlmG homolog protein 1-2, chloroplastic**	33.99	development.unspecified	Not required for the biogenesis and accumulation of native cytochrome b6 in the thylakoid membrane. Not functionally involved in the pathway for covalent binding of the c-type heme to cytochrome b6.
**Zinc finger protein WIP3**	27.3.11	RNA.regulation of transcription.C2H2 zinc finger family	Mediates and enhances the nuclear envelope docking of RANGAP proteins mediated by WIT1 and WIT2 in the undifferentiated cells of root tips.As component of the SUN-WIP-WIT2-KAKU1

	complex, mediates the transfer of cytoplasmic forces to the nuclear envelope (NE), leading to nuclear shape changes
